# Genetic Diversity and Prevalence of *Giardia duodenalis* in Qatar

**DOI:** 10.3389/fcimb.2021.652946

**Published:** 2021-05-04

**Authors:** Marwa Chourabi, Sonia Boughattas, Atiyeh M. Abdallah, Ahmed Ismail, Jerzy M. Behnke, Hesham M. Al-Mekhlafi, Marawan Abu-Madi

**Affiliations:** ^1^ Biomedical Research Center, College of Health Sciences-QU Health, Qatar University, Doha, Qatar; ^2^ Department of Biomedical Sciences, College of Health Sciences-QU Health, Doha, Qatar; ^3^ Biomedical and Pharmaceutical Research Unit-QU Health, Qatar University, Doha, Qatar; ^4^ Medical Commission, Ministry of Public Health, Doha, Qatar; ^5^ School of Life Sciences, University of Nottingham, Nottingham, United Kingdom; ^6^ Medical Research Centre, Jazan University, Jazan, Saudi Arabia; ^7^ Department of Parasitology, Faculty of Medicine and Health Sciences, Sana’a University, Sana’a, Yemen

**Keywords:** *G. duodenalis*, assemblages, multilocus genotyping, Qatar, immigrant

## Abstract

**Background:**

*Giardia duodenalis* is a common human intestinal parasite worldwide, and the causative agent of diarrhea, with the severity of disease ranging from asymptomatic to intense and debilitating infection. *G. duodenalis* is known to consist of eight genetically distinct assemblages, named from A to H. No data available on the genotypes and genetic diversity of *G. duodenalis* circulating in Qatar.

**Methods:**

We genotyped 54 human *Giardia* isolates, collected from asymptomatic immigrants in Qatar, using a multilocus genotyping (MLGs) tool. We also investigated relationships between the subjects’ genotypes and their demographic data.

**Results:**

Genomic DNA from 54 isolates were tested by PCR and sequence analysis at three loci: *glutamate dehydrogenase* (*gdh*), *β-giardin* (*bg*) and *triose phosphate* (*tpi*)). Assemblage A was identified in nine (16.67%), assemblage B in thirty (55.55%), and a mixture of assemblages A+B in fifteen (27.78%) isolates. All assemblage A isolates, genotyped in different loci, were assigned to sub-assemblage AII, and six of them had MLGs AII-1 while one new MLG was identified in two isolates. Sequences of assemblage B isolates have high level of genetic diversity and high presence of heterogeneous peaks, especially within the *gdh* gene. No significant associations between genotypes and the immigrants’ demographic data were found due to the extensive number of new variants.

**Conclusions:**

MLGs was used herein to genotype 54 immigrant *Giardia* isolates. The high level of genetic variability found in our isolates hampered MLGs determination, more investigations are now required to consolidate our findings, and to enable a comprehensive understanding of the diversity within *G. duodenalis* assemblage B isolates.

## Introduction

*Giardia duodenalis* (*G. lamblia, G. intestinalis*) is a common reported intestinal protozoan among human populations worldwide, with about 280 million people affected annually (Squire and Ryan, 2017). It accounts for more than 2.5 million annual diarrhea cases in developing countries (Thompson, 2000). Noteworthy, reporting systems do not exist in many of the countries where *G. duodenalis* is most prevalent, and/or diarrhea is not considered to be a disease locally. Giardiasis is also typically associated with asymptomatic infections that act as carriers and may remain unreported (Younas et al., 2008). Due to its high prevalence and its impact on health, giardiasis was considered as Neglected Disease by the World Health Organization in 2004 (Savioli et al., 2006). The parasite is genetically classified into eight separate divisions called ‘Assemblages’ (from A to H), which differ in their specificity for hosts (Feng and Xiao, 2011). Human infection is mainly caused by assemblages A and B (Cacciò et al., 2005). Furthermore, molecular investigations have reported sub-assemblages in assemblages A and B. Assemblage A is divided into three sub-assemblages (AI, AII, AIII) and B into two (BIII and BIV) that are genetically very similar and closely related to each other within the same assemblage (Sprong et al., 2009; Feng and Xiao, 2011).

Molecular genotyping of key genes allows tracing the sources of *Giardia’s* contamination; thereby help to understand the epidemiology of this disease agent. Single locus analysis has been used as the main tool in most molecular epidemiological studies to-date, to determine the levels of genetic variation within assemblages. However, more recently multilocus genotyping (MLGs) have become widely available and used to determine new isolates (Sulaiman et al., 2003). Three genes are used for genotyping and sub-typing of *G. duodenalis*: *glutamate dehydrogenase (gdh)*, *β-giardin (bg)* and *triose phosphate (tpi)* (Cacciò and Ryan, 2008). Moreover, a nomenclature system has been established for naming *G. duodenalis* subtypes with several subtypes (frequently named as A1–A6) have been described at each of the genetic loci (Cacciò et al., 2008; Xiao and Feng, 2017). Therefore, based on MLGs analysis of the three genetic loci, several MLGs (e.g. AI-1, AI-2, AII-1, AII-2, AII-3, AII-4, …., AII-15, AIII-1) have been described for assemblage A following a nomenclature system-derived matrix for naming (Faria et al., 2017; Naguib et al., 2018). On the other hand, due to the presence of extensive allelic sequence heterozygosity in assemblage B at the related genotyping and subtyping loci, applying this nomenclature system might be not possible for assemblage B (Feng and Xiao, 2011; Xiao and Feng, 2017). The biological and epidemiological characteristics of assemblages A and B are yet to be fully understood and virulence of these assemblages have so far turned out to be contradictory (Wielinga and Thompson, 2007; Minetti et al., 2015). Although recent reports have suggested possible associations between assemblage occurrence and the age and/or sex of subjects (Faria et al., 2017), clinical manifestations (Mohammed Mahdy et al., 2009), and/or drug sensitivity (Sahagún et al., 2008), further studies are required to confirm these associations and to enable a thorough understanding of their epidemiological significance.

Rapid socio-economic development in the state of Qatar has attract large numbers of immigrants to the country in the last two decades, mainly from developing countries. The high prevalence of intestinal parasitic infections (e.g. *G. duodenalis, Cryptosporidium, Endolimax nana*), among immigrants, has motivated the government to change the laws governing eligibility for work permits in the country. The prevalence and abundance of intestinal protozoan infections have been investigated by our research team in previously (Abu-Madi et al., 2008; Abu-Madi et al., 2013; Abu-Madi et al., 2016a; Abu-Madi et al., 2016b). Based on conventional microscopy, the overall prevalence of *G. duodenalis*, varied among newly arrived immigrants workers from 1.77% to 3.02% with the highest prevalence among immigrants arriving from Asia (3.6%) (Bonhomme et al., 2011). Unfortunately, data regarding the genetic diversity of *G. duodenalis* assemblages and sub-assemblages for immigrants in Qatar are unavailable. Therefore, our aim was to investigate the diversity and molecular variability of three key informative genes of *G. duodenalis* in samples obtained from asymptomatic immigrants referred to the medical commission of Qatar as a mandatory requirement to obtain work permit. We used MLGs to characterize the genotypes of *Giardia* isolates, and investigated the likelihood of any association between specific assemblages and the age, sex or country of origin of our participants.

## Materials and Methods

### Ethics Statement

The Medical Research Centre and Research Committee at Hamad Medical Corporation provided the ethical approval for this project [Research protocol # 16367/16 (NPRP–1556–3-313)]. Written informed consent was obtained from all participants.

### Real-Time PCR Detection

A total of 985 stool samples were collected between 2012 and 2016, from asymptomatic individuals referred for medical checkup at the Medical Commission in Qatar (Abu-Madi et al., 2008). Relevant demographic and personal details of the subjects in this study have already been fully described in previous publications (Abu-Madi et al., 2008; Abu-Madi et al., 2013). DNA extraction was performed using the QIAamp DNA stool minikit (Qiagen, Hilden, Germany). Molecular detection of *G. duodenalis* was initially carried out by real-time PCR (RT-PCR) analysis (Applied Biosystems Cycler 7500) using HotStar Taq Plus Master Mix Kit (Qiagen, Hilden, Germany). In a total reaction volume of 20 μl, 3 μl of DNA were added to 0.3 μM of forward primer (*G. intestinalis F:* 5’ GACGGCTCAGGACAACGGTT 3’), 0.3 μM of reverse primer (*G. intestinalis R:* 5’ TTGCCAGCGGTGTCCG 3’) and 0.03 μM of probe (6-FAM)-5’-CCCGCGGCGGTCCCTGCTAG-3’ (BHQ1)) targeting a 62 bp region of the small-subunit ribosomal RNA *(SSU rRNA)* of the parasite (Abu-Madi et al., 2017). The PCR cycles have an initial hold step of 2 min at 72°C and 10 min at 95°C, followed by 40 cycles of 15 sec at 95°C, 33 sec at 60°C and 30 sec at 72°C and ended by a final extension of 2 min at 72°C. Positive and negative controls were included.

### *Giardia* Genotyping

*G. duodenalis* samples tested positive were analyzed by MLGs at the *glutamate dehydrogenase (gdh)*, *β-giardin (bg)* and *triosephosphate isomerase (tpi)* loci. A semi-nested PCR amplified a 432 bp fragment of the *gdh* gene using the primers sets GDHeF and GDHiR in the primary PCR, and GDHiF and GDHiR in the secondary reaction [primer sequences are given in **Table 1** (Abu-Madi et al., 2017)]. Amplification reactions were processed as following: 1 cycle of 3 min at 95°C, then 35 cycles of 30 sec at 95°C, 33 sec at 55°C and 1 min at 72°C, and a final extension of 10 min at 72°C. For the *bg* gene, two-step nested PCRs were performed to amplify a 753 bp fragment during the primary PCR and a 511 bp fragment during the secondary reaction. The two sets of primers used were G7_F and G759_R and bg-GiarF and bg-GiarR, as external and internal primers, respectively [primers are listed in **Table 1** (Verweij et al., 2003)]. The primary PCR reaction conditions are: 1 cycle of 95°C for 7 min, followed by 35 cycles of 95°C for 30 sec, 65°C for 30 sec, and 72°C for 1 min with a final extension of 72°C for 7 min. Similar conditions were followed for the secondary PCR except for the annealing temperature which was 55°C. *tpi* gene were amplified by two-step nested PCRs, a 605 bp fragment of the *tpi* gene in the primary reaction and a 532 bp fragment in the secondary PCR using the two primer pairs: AL-3543 with AL-3546 and AL-3544 with AL-3545 [**Table 1** (Lalle et al., 2005)]. The PCR reaction conditions were the same and as follow: 1 cycle of 94°C for 5 min, followed by 35 cycles of 94°C for 45 sec, 50°C for 45 sec, and 72°C for 1 min with a final extension of 72°C for 10 min.

**Table 1:** Primers, targets, sizes and assay type used for *G. duodenalis* genotyping.

Primer name	Sequence (5’-3’)	Gene	Size (bp)	Assay type	References
**GDHeF**	TCAACGTYAAYCGYGGYTTCCGT	*GDH*	432	Semi nested PCR, Sequencing	Monis and Thompson, 2003
**GDHiF**	CAGTACAACTCYGCTCTCGG				
**GDHiR**	GTTRTCCTTGCACATCTCC				
**G7**	AAGCCCGACGACCTCACCCGCAGTGC	*BG*	753	Nested PCR, Sequencing	Cacciò and Sprong, 2010
**G759**	GAGGCCGCCCTGGATCTTCGAGACGAC				
**GiarF**	GAACGAACGAGATCGAGGTCCG		511		
**GiarR**	CTCGACGAGCTTCGTGGTT				
**AL3543**	AAATIATGCCTGCTCGTCG	*TPI*	605	Nested PCR, Sequencing	Geurden et al, 2009
**AL3546**	CAAACCTTITCCGCAAACC				
**AL3544**	CCCTTCATCGGIGGTAACTT		532		
**AL3545**	GTGGCCACCACICCCGTGCC				

The PCR reactions for the three investigated loci were carried out in an Applied Biosystems 9700 thermal cycler and the amplified fragments were visualized on 1% agarose gels and stained with SYBR Safe (Invitrogen, USA). PCR products were purified using QIAquick gel extraction kit (Qiagen, Hilden, Germany). The obtained amplicons were sequenced at MCLAB facilities (Molecular Cloning Laboratories- San Francisco, USA) in both directions. The sets of primers used for direct sequencing of *gdh*, *bg* and *tpi* genes were GDHiF and GDHiR, GiarF and GiarR, and AL5344 and AL3545, respectively (**Table 1**).

### Data Analyses

Sequencing data were analysed using the FinchTV 1.4 sequence analysis program (https://finchtv.software.informer.com/1.4/) and aligned and edited using the BioEdit software (http://www.mbio.ncsu.edu/BioEdit/bioedit.html). BLAST was used to compare DNA sequences with sequences already deposited in the GenBank database (https://blast.ncbi.nlm.nih.gov/Blast.cgi). ClustalW in BioEdit software was used to perform multiple alignment analyses to determine *G. duodenalis* assemblages, sub-assemblages and subtypes. Identification of MLGs within *G. duodenalis* assemblage A was performed based on multilocus sequence typing (MLST) analysis for nucleotide sequences at the *tpi*, *bg*, and *gdh* loci, using the established nomenclature system (Cacciò et al., 2008). With regards assemblage B, the presence of extensive allelic sequence heterozygosity at these *tpi*, *bg*, and *gdh* loci (Roellig et al., 2015; Xiao and Feng, 2017), it may be problematic to subtype assemblage B specimens based on MLST for sequence analysis of these loci. However, identification of sub-assemblages was performed for isolates that found identical (100%) and isolates with a single heterozygous peak when aligned with some representative reference sequences. Nonetheless, isolates with apparent presence of more than one heterozygous peak at any of the loci during DNA sequencing were excluded in the MLG analysis of sequence data; and therefore, reported as “assemblage B” (Wang et al., 2019). Representative sequences from our isolates have been deposited in GenBank under accession numbers reported in the results section.

## Results

### Participants Demographic Data

Of the 985 subjects, 54 were confirmed positive for *G. duodenalis* by RT-PCR (5.48%). Demographic data for the 54 participants are available in **Supplementary Table 1**, except for only one subject (S26). The age of the 53 individuals infected with *G. duodenalis* ranged from 19 to 44 years; four were females and 49 were males. Only two males, in our study, were from Africa and the reset were Asian. The distribution of *Giardia* sub-assemblages in relation to demographic data is shown in **Supplementary Table 1**.

### *Giardia duodenalis* Genotypes and Subtypes

For the 54 subjects, the *gdh*, *bg* and *tpi* genes were successfully amplified in 96.29%, 100% and 94.44% respectively. PCR analyses yielded the expected amplicons for *gdh, bg* and *tpi* loci in all isolates, and sequence alignment scrutiny of the obtained PCR products, revealed assemblage A in 9 isolates (16.67%), assemblage B in 30 isolates (55.55%) and mixed assemblages (A+B) in 15 isolates (27.78%) (**Table 2**).

**Table 2:** Assemblages and sub-assemblages of *G. duodenalis* isolates characterized by sequencing in this study.

**No. positive**	Isolates ID	Sequence data			
		*gdh*	*bg*	*tpi*	MLG (*gdh+bg+tpi*)
8	S15, S17, S25, S26, S28, S33, S36 and S52	Assemblage A: AII	Assemblage A: AII	Assemblage A: AII	Assemblage A: AII
1	S22	Assemblage A: AII	Assemblage A: AII	NA	Assemblage A: AII
6	S6, S8, S13, S34, S48 and S49	Assemblage B	Assemblage B	Assemblage A: AII	Assemblage A + B
6	S7, S16, S23, S27, S39 and S40	Assemblage A: AII	Assemblage B	Assemblage A: AII	Assemblage A + B
1	S45	Assemblage B	Assemblage A: AII	Assemblage B	Assemblage A + B
1	S18	NA	Assemblage B	Assemblage A: AII	Assemblage A + B
1	S31	Assemblage A: AII	Assemblage B	NA	Assemblage A + B
2	S44 and S50	Assemblage B	Assemblage B	NA	Assemblage B
1	S24	NA	Assemblage B	Assemblage B	Assemblage B
27	S1-S5, S9-S12, S14, S19-S21, S29, S30, S32, S35, S37, S38, S41-S43, S46, S47, S51, S53 and S54	Assemblage B	Assemblage B	Assemblage B	Assemblage B

NA, Not Amplified.

### Molecular Typing of Assemblage A

Out of the 52 isolates of *G. duodenalis* amplified at the *gdh* locus, 16 (30.76%) were classified as assemblage A (**Table 2**). All these isolates were assigned to the sub-assemblage AII and exhibited 100% similarity to the reference sequence AII (accession no. KY444774.1), and they were all identical to the A2 subtype when aligned with some respective reference sequences in GenBank (e.g. accession no: KY444774.1 and L40510). A representative sequence of these isolates has been deposited in GenBank under the accession no: MN867384. Multiple sequence alignment analysis showed that among 54 samples sequenced at the *bg* locus, ten (18.51%) were assigned to assemblage A and were typed as AII. They were all 100% identical to the reference sequence AII (accession no: FN377868.1) and they were all typed as A2 when aligned with some reference sequences from the GenBank database. A representative sample of these isolates is available in GenBank under the accession no: MN867385. Overall, there was no evidence of heterogeneous positions at both *gdh* and *bg* loci.

At the *tpi* locus, alignment analysis revealed that 21 isolates (41.18%) from the 51 genotyped isolates were typed as sub-assemblage AII when aligned with reference sequence AII (accession no: KR075936.1). Nineteen of these fully matched (100% similarity) the reference sequence and they were identical to the A2 subtype (**Table 3**). On the other hand, there was a one nucleotide substitution from G to A in two isolates (S15 and S52) at position 347 when aligned with reference sequence KR075936.1 (corresponded to position 445 of the *tpi* gene), and these isolates were identified as a novel subtype A2 (**Table 3**). Representative sequences of these two different types of isolates have been submitted to GenBank with accession no: MN867386 and MN867387, respectively.

**Table 3:** Heterozygous positions within tpi gene among *G. duodenalis* sub-assemblage. AII isolates of this study with reference sequences obtained from GenBank.

Reference/Isolate ID	GenBank accession no.	No. of isolates	Subtype	Nucleotide position from the start of the *tpi* gene			
**Reference sequences**				129	349	399	445
A2	KR075936.1	–	A2	**C**	**G**	**T**	**G**
JH	U57897	–	A2	.	.	.	.
Ad-2	AF069557	–	A2	.	.	.	.
TB11.6	KC313923	–	A2	.	.	.	.
INI 44	KF922901	–	Novel A2^**^	.	**A**	.	.
WB	L02120	–	A1	**T**	.	**C**	.
**Study isolates**							
S6, S7, S8, S13, S16, S17, S18, S23, S25, S26, S27, S28, S33, S34, S36, S39, S40, S48 and S49	MN867386^*^	19	A2	.	.	.	.
S15 and S52	MN867387^*^	2	Novel A2^†^	.	.	.	**A**

Nucleotide substitution is in bold and in capital letter, dots indicate identity to the reference sequence (accession number http://www.KR075936.1). ^*^ Representative GenBank accession numbers for study isolates. ^**^A new sequence type identified by Faria et al. (2017). ^†^A new sequence type identified in the present study.

### MLG of Isolates Representing Assemblage A

In total, multiple alignment analysis revealed that 16, 10 and 21 isolates from partial amplification of the *gdh, bg* and *tpi* loci respectively, were typed as sub-assemblage AII. Noteworthy, of the three loci (*gdh/bg/tpi*) only eight isolates (S15, S17, S25, S26, S28, S33, S36 and S52) were assigned to AII based on sequence analysis, whereas the remaining isolates comprised mixed genotypes of assemblages A and B (**Table 2**). As regards subtypes MLG, a previously identified MLG AII-1 (profile: A2/A2/A2) was found in six isolates, whereas a new AII MLG (profile: A2/A2/novel A2) was identified in two isolates (**Table 4** and **Supplementary Table 1**).

**Table 4:** Multilocus sequence types of *G. duodenalis* sub-assemblage AII isolates.

Isolate ID	No. positive	Subtype (Reference sequence accession no.)			MLGs (*gdh+bg+tpi*)
		*gdh*	*bg*	*tpi*	
S17, S25, S26, S28, S33 and S36	6	A2 (L40510)	A2 (FJ560582)	A2 (KR075936)	AII-1
S15 and S52	2	A2 (L40510)	A2 (FJ560582)	New A2 (MN867387)*	New AII
S22	1	A2 (L40510)	A2 (FJ560582)	–	–
S7, S16, S23, S27, S39 and S40	6	A2 (L40510)	–	A2 (KR075936)	–
S6, S8, S13, S34, S48 and S49	6	–	–	A2 (KR075936)	–
S18	1	–	–	A2 (KR075936)	–
S45	1	–	A2 (FJ560582)	–	–
S31	1	A2 (L40510)	–	–	–

*A new sequence type identified in the study.

### Molecular Typing of Assemblage B

DNA sequence analyses showed that at the *gdh* locus, 36/52 isolates were characterized as assemblage B and were first aligned with reference sequences: JQ700429.1 and EU834843.1 representing the sub-assemblages BIII and BIV, respectively (**Table 5**). Although alignment analyses showed that all these 36 isolates were similar to sub-assemblage BIII (accession no: JQ700429.1), they could not be assigned unequivocally as sub-assemblage BIII because of the number of variations identified (from 2 to 8 variations). Therefore, they are referred to as assemblage B, with nucleotides substitutions and variation with reference sequences are carefully described. Most samples (e.g. S1, S11, S45, S32, S48) exhibited heterozygous peaks in chromatogram profiles. In fact, all these five isolates showed heterozygous peaks at positions: T235C, T331C and T352C. Moreover, isolates S1, S11, S32 and S48 revealed heterozygous peaks at position T163C, and isolates S32 and S48 showed heterozygous peaks at positions T253C and T359C. Isolate S45 also exhibited a heterozygous peak at position T359C (**Table 5**).

**Table 5:** Single nucleotide polymorphisms detected in *G. duodenalis* assemblage B isolates at the gdh locus.

Isolates/ Sequence	Nucleotide at position of reference sequence BIII_JQ700429.1														No. of differences with reference	Accession No.
	157	158	163	166	202	205	212	232	235	253	304	331	352	359		
**BIII_JQ700429.1**	C	A	T	G	T	G	C	C	T	T	C	T	C	C		
**S12**	.	.	.	.	.	.	**T**	.	.	.	.	.	.	**Y**	**2**	**MN867388**
**S5**	.	.	.	**R**	.	.	**T**	.	.	.	.	.	.	**Y**	**3**	**MN867389**
**S10**	.	.	**C**	**R**	.	.	**T**	.	.	.	.	.	.	.	**3**	**MN867390**
**S44**	**T**	.	.	.	.	.	**T**	.	.	.	.	.	.	**T**	**3**	**MN867391**
**S34**	**T**	.	**C**	**A**	.	.	**T**	.	.	.	.	.	.	.	**4**	**MN867392**
**S35**	.	.	**Y**	.	.	.	**T**	.	.	.	.	**C**	**Y**	.	**4**	**MN867393**
**S42**	.	.	.	.	.	.	**T**	.	**C**	.	.	**C**	**Y**	.	**4**	**MN867394**
**S6**	.	**G**	**C**	.	.	.	**T**	.	**C**	**C**	.	.	.	.	**5**	**MN867395**
**S1**	.	.	**Y**	.	.	.	**T**	.	**Y**	.	.	**Y**	**Y**	.	**5**	**MN867396**
**S11**	.	.	**Y**	.	.	.	**T**	.	.	.	.	**Y**	**Y**	.	**5**	**MN867397**
**S19**	.	.	.	.	**Y**	.	**T**	.	.	.	.	**C**	**Y**	**Y**	**5**	**MN867398**
**S30**	.	.	**C**	.	.	.	**T**	.	**C**	.	.	**C**	**T**	.	**5**	**MN867399**
**S46**	.	.	.	.	.	.	**T**	.	**C**	**Y**	.	**C**	**T**	.	**5**	**MN867400**
**S54**	.	.	**Y**	.	.	.	**T**	.	**C**	.	.	**C**	**Y**	.	**5**	**MN867401**
**S2**	.	.	**C**	.	.	.	**T**	.	**C**	**C**	.	**Y**	**T**	.	**6**	**MN867402**
**S3, S29, S38 and S41**	.	.	**C**	.	.	.	**T**	.	**C**	**Y**	.	**C**	**T**	.	**6**	**MN867403**
**S4**	.	.	**Y**	.	.	.	**T**	.	**C**	**C**	.	**C**	**T**	.	**6**	**MN867404**
**S8**	.	.	**Y**	.	.	.	**T**	.	**C**	**Y**	.	**C**	**T**	.	**6**	**MN867405**
**S9**	.	.	**C**	.	.	.	**T**	.	**C**	**C**	.	**C**	**Y**	.	**6**	**MN867406**
**S13**	.	.	.	.	.	.	**T**	**Y**	**C**	**Y**	.	**C**	**T**	.	**6**	**MN867407**
**S14**	.	.	**C**	.	.	.	**T**	.	**C**	**C**	.	**C**	**T**	.	**6**	**MN867408**
**S37**	.	.	**Y**	.	.	.	**T**	.	**C**	**C**	.	**C**	**T**	.	**6**	**MN867409**
**S43**	.	.	**C**	.	.	.	**T**	.	**C**	**C**	.	**C**	**T**	.	**6**	**MN867410**
**S45**	.	.	**C**	.	.	.	**T**	.	**Y**	.	.	**Y**	**Y**	**Y**	**6**	**MN867411**
**S47**	.	.	**C**	.	.	.	**T**	.	**C**	**C**	.	**C**	**T**	.	**6**	**MN867412**
**S53**	.	.	**Y**	.	.	.	**T**	.	**C**	**Y**	.	**C**	**Y**	.	**6**	**MN867413**
**S21**	.	.	**Y**	.	.	.	**T**	.	**C**	**C**	**T**	**C**	**T**	.	**7**	**MN867414**
**S32**	.	.	**Y**	**R**	.	.	**T**	.	.	**Y**	.	**Y**	**Y**	**Y**	**7**	**MN867415**
**S48**	.	.	**Y**	.	.	.	**T**	.	**Y**	**Y**	.	**Y**	**Y**	**Y**	**7**	**MN867416**
**S49**	.	.	**Y**	.	.	.	**T**	**Y**	**C**	**Y**	.	**C**	**T**	.	**7**	**MN867417**
**S51**	.	.	**Y**	.	.	**A**	**T**	.	**C**	**Y**	.	**C**	**T**	.	**7**	**MN867418**
**S20**	.	.	**Y**	.	.	**A**	**T**	**Y**	**C**	**Y**	.	**C**	**T**	.	**8**	**MN867419**
**S50**	.	.	**Y**	.	.	**A**	**T**	**Y**	**C**	**C**	.	**C**	**T**	.	**8**	**MN867420**

Nucleotide substitutions are in bold and in capital letters, dots indicate identity to the reference sequence (BIII_JQ700429.1), heterozygous positions are in bold and indicated by standard IUPAC codes (Y: T/C and R: G/A).

Interestingly, all our isolates displayed a novel substitution at position 212 (BIII_JQ700429.1, 212C>T) not previously reported (**Table 5**). In addition, all isolates showed crucial diversity with each other and with previously published sequences (more than three new variations, **Table 5**). In fact, only four isolates (S3, S29, S38 and S41) had the same genetic profile but differed by 6 nucleotides with the BIII_JQ700429.1 sequence. The remaining isolates differed by 2–8 nucleotides. Accession numbers of all variants, already submitted to GenBank, are indicated in **Table 5**.

Similarly, among 54 samples genotyped at the *bg* gene, 44 were clearly classified as assemblage B. The raw data sequence reads were first aligned with reference sequences BIII_KJ188086.1 and BIV_KM926514.1 among others that were available in GenBank. Multiple sequence alignment analysis revealed that only one isolate (S43) showed complete identity with published sequence BIV_KM926514.1 and thus was assigned as sub-assemblage BIV (**Table 6**). Moreover, twelve isolates (S1, S2, S4, S9, S11, S23, S40, S41, S42, S46, S50 and S54) differed with the BIII_KJ188086.1 sequence by only one nucleotide at position 130 (130 T>C) (**Table 6**) and were therefore subtyped as BIII. Likewise, nucleotide sequences of isolates S12, SS14, S18, S24 and S53 revealed a one nucleotide difference at position 16 (16T>C) compared to sub-assemblage BIV_KM926514.1, suggesting an assignment to BIV.

**Table 6:** Single nucleotide polymorphisms detected within *G. duodenalis* assemblage B isolates at the bg locus.

Isolates/ Sequence	Nucleotide at position of reference sequences BIII_KJ188086.1 and BIV_KM926514.1										No. of differences with reference sequences*		Accession No.
	16	34	88	91	130	148	160	185	212	370		
**BIII_KJ188086.1**	C	A	G	T	T	C	C	A	A	T	**BIII**	**BIV**	
**BIV_KM926514.1**	T	A	G	T	C	C	T	A	A	T			
**S43**	**T**	.	.	.	**C**	.	**T**	.	.	.	3	0	MN867443
**S1, S2, S4, S9, S11, S23, S40, S41, S42, S46, S50 and S54**	**C**	.	.	.	**C**	.	**C**	.	.	.	1	2	MN867444
**S12, S14, S18, S24 and S37**	**C**	.	.	.	**C**	.	**T**	.	.	.	2	1	MN867445
**S3, S13, S19, S20, S27, S34, S35, S39, S44, S47, S48, S49, S51 and S53**	**C**	.	.	.	**C**	.	**Y**	.	.	.	2	2	MN867446
**S6 and S31**	**C**	.	.	.	**C**	.	**T**	.	.	**C**	3	2	MN867447
**S7**	**C**	**R**	.	.	**C**	.	**C**	.	.	.	2	3	MN867448
**S8**	**C**	.	.	.	**C**	.	**Y**	.	.	**C**	3	3	MN867449
**S16 and S30**	**C**	**G**	.	.	**C**	.	**T**	.	.	.	3	2	MN867450
**S21 and S29**	**C**	.	**A**	.	**C**	.	**C**	.	.	.	2	3	MN867451
**S32**	**C**	.	.	**Y**	**C**	.	**Y**	.	.	.	3	3	MN867452
**S5**	**C**	**G**	.	.	**C**	.	**T**	.	.	**C**	4	3	MN867453
**S10**	**C**	**G**	.	.	**C**	.	**Y**	.	.	**C**	4	4	MN867454
**S38**	**C**	**R**	.	.	**C**	.	**Y**	**R**	**R**	**Y**	6	6	MN867455

Nucleotide substitutions are in bold and in capital letters, dots indicate identity to both reference sequences (BIII_KJ188086.1 and BIV_KM926514.1), heterozygous positions are in bold and indicated by standard IUPAC codes (Y: T/C and R: G/A). *Compared to reference sequences BIII_KJ188086.1 and BIV_KM926514.1.

On the other hand, the sequences of the remaining isolates (26 isolates) corresponded to a mixture of sub-assemblages BIII and BIV when compared with the BIII_KJ188086.1 and BIV_KM926514.1 reference sequences, with multiple SNPs and heterozygous peaks also characterized these sequences. Therefore, these isolates were excluded in the MLG analysis of sequence data and therefore reported as assemblage B without inferring the sub-assemblages. A group of 14 isolates (S3, S13, S19, S20, S27, S34, S35, S39, S44, S47, S48, S49, S51 and S53) had one SNP at position 16 (16T>C; BIV_KM926514.1) compared to BIV, one SNP at position 130 (130T>C; BIII_KJ188086.1) compared to BIII and a heterozygous nucleotide at position 160 (C160T) (**Table 6**). In addition to the SNP at position 16 (16T>C; BIV_KM926514.1), the nucleotide sequence of isolates S6 and S31 revealed a novel substitution of T to C at position 370 (370T>C; BIV_KM926514.1). Similarly, two isolates S16 and S30 exhibited a novel nucleotide variation at position 34 (34A>G; BIV_KM926514.1) together with the cited SNP at position 16 (16T>C; BIV_KM926514.1). However, isolate S5, differed by two novel substitutions at positions 34 (34A>G; BIV_KM926514.1) and 370 (370T>C; BIV_KM926514.1) in addition to the change from T to C at position 16 (16T>C; BIV_KM926514.1) (**Table 6**). Sequence reads of isolate S7 showed, in addition to the substitution of T to C at position 130 (130T>C; BIII_KJ188086.1), one heterozygous peak at position 34 (A34G; BIII_KJ188086.1). Furthermore, two isolates S21 and S29, which were distinct from BIII by the substitution of T to C at position 130 (130T>C; BIII_KJ188086.1), both showed one novel variation of G to A at position 88 (88G>A; BIII_KJ188086.1). Isolates S8 and S32 both showed a mixture of BIII/BIV, but S8 had a novel substitution of T to C at position 370 (370T>C; BIV_KM926514.1; BIII_KJ188086.1) while S32 indicated a heterozygous peak at position 91 (T91C; BIV_KM926514.1; BIII_KJ188086.1). Isolates S10 and S38 differed by more than three nucleotides with reference sequences (either BIII_KJ188086.1 or BIV_KM926514.1). All the SNPs and heterozygous positions detected in *G. duodenalis* assemblage B isolates at the *bg* locus, as well as accession numbers of isolate sequences submitted to GenBank are summarized in **Table 6**.

At the *tpi* locus, of 51 isolates 30 were classified as assemblage B. Alignment analysis was carried out using the reference sequences available in GenBank: BIII_AF069561.1 and BIV_AF069560.1 (**Table 7**). Multiple sequence alignment analysis of eight isolates (S9, S19, S24, S29, S32, S35, S43 and S51) showed full match with reference sequence (BIII_AF069561.1) and allowed us to assign them to sub-assemblage BIII. Sequence analysis of seven isolates (S47, S5, S37, S11, S20, S10 and S4) indicated that they could also be assigned to BIII since they each differed from BIII_AF069561.1 by only one novel substitution at positions 21, 54, 63, 78, 136, 220 and 255, respectively (**Table 7**). Chromatogram readings identified single SNPs in isolates S38, S42 and S46, presenting as heterozygous peaks at positions 137, 306 and 63 respectively (BIII_AF069561.1). These three isolates were assigned to BIII. The sequences of two isolates (S2 and S30) revealed one heterozygous peak at position 111 (G111A; BIII_AF069561.1, **Table 7**) corresponding to sub-assemblages BIII. On the other hand, the sequences of the remaining 10 isolates showed more than one heterozygous peak when compared with the BIII_AF069561.1 and BIV_AF069560.1 sequences; therefore, sub-assemblages could not be inferred. Isolates S14, S21 and S41 showed between 2–3 heterozygous peaks at different positions compared to both reference sequences, BIV_AF069560.1 and BIII_AF069561.1 (**Table 7**). Likewise, three isolates S1, S3 and S53 differed by 3 nucleotides with BIII_AF069561.1 and showed heterozygous peaks at different positions. Isolates S12 and S45 showed two novel heterozygous peaks identified in each of the following: S12: C141T and C339T; S45: C54T and G146A; BIV_AF069560.1; BIII_AF069561.1). Sequence analyses of isolates S31 and S54 revealed that they differed from the reference sequences BIV_AF069560.1 and BIII_AF069561.1 in GenBank by 6 and 7 heterozygous peaks, respectively (**Table 7**). All the nucleotide variations at the *tpi* locus detected in *G. duodenalis* assemblage B isolates are described in **Table 7**. Representative sequences have been submitted to GenBank and accession numbers are also given in **Table 7**.

**Table 7:** Single nucleotide polymorphisms detected within *G. duodenalis* assemblage B isolates at the tpi locus.

Isolates/Sequence	Nucleotide at position of reference sequences BIII_AF069561.1																								No. of differences with reference	Accession No.
	6	10	21	42	54	63	66	69	75	78	111	136	137	141	146	159	172	181	198	220	255	302	306	339		
**BIII_AF069561.1**	G	G	T	C	T	G	C	C	G	T	G	A	C	C	A	C	C	A	A	A	G	A	C	T		
**BIV_AF069560.1**	G	G	T	C	T	G	T	T	G	T	A	A	C	C	A	C	C	A	A	A	G	A	C	T		
**S9, S19, S24, S29, S32, S35, S43 and S51**	.	.	.	.	.	.	.	.	.	.	.	.	.	.	.	.	.	.	.	.	.	.	.	.	**0**	MN867421
**S47**	.	.	**C**	.	.	.	.	.	.	.	.	.	.	.	.	.	.	.	.	.	.	.	.	.	**1**	MN867422
**S5**	.	.	.	.	**C**	.	.	.	.	.	.	.	.	.	.	.	.	.	.	.	.	.	.	.	**1**	MN867423
**S37**	.	.	.	.	.	**A**	.	.	.	.	.	.	.	.	.	.	.	.	.	.	.	.	.	.	**1**	MN867424
**S11**	.	.	.	.	.	.	.	.	.	**C**	.	.	.	.	.	.	.	.	.	.	.	.	.	.	**1**	MN867425
**S20**	.	.	.	.	.	.	.	.	.	.	.	**G**	.	.	.	.	.	.	.	.	.	.	.	.	**1**	MN867426
**S10**	.	.	.	.	.	.	.	.	.	.	.	.	.	.	.	.	.	.	.	**G**	.	.	.	.	**1**	MN867427
**S4**	.	.	.	.	.	.	.	.	.	.	.	.	.	.	.	.	.	.	.	.	**A**	.	.	.	**1**	MN867428
**S2 and S30**	.	.	.	.	.	.	.	.	.	.	**R**	.	.	.	.	.	.	.	.	.	.	.	.	.	**1**	MN867429
**S38**	.	.	.	.	.	.	.	.	.	.	.	.	**Y**	.	.	.	.	.	.	.	.	.	.	.	**1**	MN867430
**S42**	.	.	.	.	.	.	.	.	.	.	.	.	.	.	.	.	.	.	.	.	.	.	**Y**	.	**1**	MN867431
**S46**	.	.	.	.	.	**R**	.	.	.	.	.	.	.	.	.	.	.	.	.	.	.	.	.	.	**1**	MN867432
**S14**	.	.	.	.	.	.	**T**	.	.	.	.	.	.	.	.	.	.	**G**	.	.	.	.	.	.	**2**	MN867433
**S21**	.	.	.	.	.	.	**T**	**Y**	.	.	.	.	.	.	.	.	.	.	.	.	.	.	.	.	**2**	MN867434
**S41**	.	.	.	.	.	.	**T**	.	.	.	.	.	.	.	.	**T**	.	.	.	.	.	.	.	.	**2**	MN867435
**S1**	.	.	.	.	.	.	**Y**	**Y**	.	.	**R**	.	.	.	.	.	.	.	.	.	.	.	.	.	**3**	MN867436
**S3**	.	.	.	.	.	.	**T**	**T**	.	.	**R**	.	.	.	.	.	.	.	.	.	.	.	.	.	**3**	MN867437
**S53**	.	.	.	.	.	.	**Y**	**Y**	**R**	.	.	.	.	.	.	.	.	.	.	.	.	.	.	.	**3**	MN867438
**S12**	.	.	.	.	.	.	**T**	**T**	.	.	**R**	.	.	**Y**	.	.	.	.	.	.	.	.	.	.	**4**	MN867439
**S45**	.	.	.	**Y**	.	.	**Y**	**Y**	.	.	.	.	.	.	**R**	.	.	.	.	.	.	.	.	**Y**	**5**	MN867440
**S31**	.	.	.	.	.	.	**Y**	**Y**	.	.	.	.	.	.	.	.	**Y**	**R**	**R**	.	.	.	.	.	**5**	MN867441
**S54**	**R**	**R**	.	**Y**	.	.	**Y**	**Y**	.	.	**R**	.	.	.	.	.	.	.	.	.	.	**R**	.	.	**7**	MN867442

Nucleotide substitutions are in bold and in capital letters, dots indicate identity to the reference sequence (BIII_AF069561.1) heterozygous positions are in bold and indicated by standard IUPAC codes (Y: T/C and R: G/A)

### MLG of Isolates Representing Assemblage B

When analyzing MLGs of the 30 isolates belonging to assemblages B, the sequences revealed wide genetic diversity and many heterogeneous positions within each gene fragment, especially in the *gdh* gene (**Supplementary Table 1**). All the isolates, sequenced at the *gdh* locus displayed high intra-isolate sequence heterogeneity and showed a number of polymorphic positions. Even though there were isolates that were clearly assigned either to sub-assemblages BIII or BIV at *tpi* or *bg* loci, respectively. The genetic heterogeneity found at the *gdh* locus prevented the unequivocal assignment of these isolates to sub-assemblages and subtypes; therefore, these isolates were reported as assemblage B without inferring the sub-assemblages or subtypes. The genotypes of each isolate in different loci are shown in **Supplementary Table 1**.

## Discussion

Genotypes of *Giardia* isolates from 54 asymptomatic immigrants in Qatar were characterized in this study. We used MLGs based on three genetic loci (*gdh, bg and tpi*), and correlated genotyping results to the demographic data of the immigrants such as age, gender and country of origin. Using multiple sequence alignment analyses we found assemblages A and B in 16.66% (9/54) and 55.55% (30/54) of samples, respectively. Mixed A and B assemblages present in 27.77%of cases (15/54). The increased percentage of assemblage B over A is in line with the reports from previous studies (van der Giessen et al., 2006; Hijjawi et al., 2016; Nunes et al., 2018). Although most studies have reported assemblage B to be more prevalent in both developed and developing countries (Costache et al., 2020; Messa et al., 2021), other studies have found a predominance of assemblage A (Thompson, 2000; Asher et al., 2016). It was suggested that the relatively high percentage of assemblage B may be attributable to the technical tools used to detect *G. duodenalis.* In fact, PCR sequence analysis based studies have shown contradictory results regarding the predominance of assemblages A or B, however, studies that used either RT-PCR or microscopy based techniques have reported a predominance of assemblage B over A (Thompson, 2004; Wielinga and Thompson, 2007).

Assemblage A is divided into sub-assemblages (AI, AII and AIII) based on polymorphisms reported at 23 positions (Feng and Xiao, 2011). In our study, there was 100% concordance between loci (e.g. *gdh + bg + tpi*) in assignment to assemblage A and to sub-assemblage AII as all the nine isolates were typed to sub-assemblage AII. Previous studies showed that AII is the predominant sub-assemblage in humans (Thompson, 2000; Haque et al., 2005). Sub-assemblages AI and AII are reported in human and animals but predominance of AII in humans (Delport et al., 2014). Since sub-assemblage AII is also reported in animals (Ryan and Cacciò, 2013), it is possible that zoonotic transmission of AII is involved but further investigations are required to clarify this issue. The presence of only sub-assemblage AII in our data could be explained by the fact that all individuals were asymptomatic, and is in line with a previous study that correlated sub-assemblage AII with asymptomatic cases (Thompson, 2000). In addition, our findings showed that six of these isolates were identified as AII-1 that corresponds to a subtype profile A2/A2/A2 at the three loci. In addition, the sequence of isolates S15 and S52 was classified as a novel variant of subtype A2 since a single point mutation is sufficient for the description of a new subtype (Sprong et al., 2009; Faria et al., 2017). These findings are consistent with previous reports that A2 subtype is the main subtype found in humans in many countries (Wang et al., 2018; Wang et al., 2019). Host adaption at the genotype and subtype levels has been suggested (Xiao and Feng, 2017); however, influence of assemblages and subtypes on clinical phenotypes are still controversial.

Sub-assemblages BIII and BIV in assemblage B have been described (Feng and Xiao, 2011). The assemblage B isolates typed at the *bg* locus were distributed as follows: 12 isolates were considered to be BIII, 6 as BIV, and no clear genotypes could be inferred in 25 isolates which were reported as assemblage B. Assemblage B isolates genotyped at the *tpi* gene were mainly typed as BIII (*n*=20), and 11 isolates were identified just as assemblage B due to high level of heterogeneity. The high prevalence of sub-assemblage BIII in our isolates at the *tpi* locus may suggest a zoonotic transmission of *Giardia* parasites. However, sequence analysis of assemblage B isolates at the *gdh* locus showed a high genetic variability preventing reliable assignment to existing sub-assemblages despite the concordance with BIII. With the exception of isolate S24 (not amplified at *gdh*), all the other isolates were not assigned to sub-assemblages B by MLGs, due to the frequent occurrence of heterogeneous variations at the *gdh* locus. Lack of concordance in the assignment to assemblage B and sub-assemblages were documented in several other studies (Cacciò et al., 2005; Ryan and Cacciò, 2013; Gasparinho et al., 2017). This lack of concordance might be explained by either mixed infections or recombination events (Xiao and Feng, 2017).

Similar to some previous studies mixed A+ B assemblage infections were identified in 15 of our isolates and all of them were not assigned to specific sub-assemblages or subtypes due to the polymorphic positions at the *gdh* locus (Haque et al., 2005). Genotyping of *G. duodenalis* using either conventional PCR (Kohli et al., 2008) or RT-PCR (Monis and Thompson, 2003) has shown a much higher prevalence of mixed infection in humans compared to what has been reported. This might be due to the presence of genetically different cysts or different alleles in the nuclei of a single cyst, called allelic sequence heterozygosity (Sulaiman et al., 2003). It is worth mentioning that most of the genotyping analyses that reported a high prevalence of mixed infections were based on cyst isolated DNA. Indeed, we used DNA extracted from stool samples that might have contained cysts. A frequent number of heterogeneous positions have been reported in studies performed in endemic areas (Thompson, 2004; Huey et al., 2013; Ryan and Cacciò, 2013). This is likely due to an increased rate of infection, and supports the theory of mixed infections that can occur at the inter-assemblages and intra-assemblages levels (Sulaiman et al., 2003). The origin of heterozygous peaks in our study could be resolved in experiments carried out using a single cyst, or clones derived from a single cell. However, genotyping results derived from single cysts and single trophozoites of assemblage B have been found to be inconclusive (Geurden et al., 2009; Cacciò and Sprong, 2010). Further investigations are required to understand the occurrence of mixed infection.

We also attempted to correlate our genotyping findings with the available demographic data of our subjects, however because it was not possible for us to determine the exact MLGs in all isolates (especially regarding assemblage B), associations between genotypes and patient demographic data were inconclusive. The large number of different countries from which our sample donors were originated might explain the high prevalence of genetic diversity found in our isolates.

## Conclusion

We carried out herein MLGs for the three genetic markers *gdh, bg* and *tpi*, which at present are still regarded as the best choice for *Giardia* genotyping. Assemblage B was found to be more prevalent than assemblage A. However, the extensive variations seen in assemblage B prevented the determination of MLGs and reliable assignment of the isolates to known accepted sub-assemblages. Further studies are recommended on a larger number of individuals as well as animals and environmental samples to enable better understanding of transmission dynamics and genetic diversity within assemblages of *G. duodenalis*.

## Data Availability Statement

The raw data supporting the conclusions of this article will be made available by the authors, without undue reservation.

## Ethics Statement

The studies involving human participants were reviewed and approved by The Medical Research Centre and Research Committee at Hamad Medical Corporation. The patients/participants provided their written informed consent to participate in this study.

## Author Contributions

Conceptualization, MA-M. Data curation, MC and SB. Formal analysis, MC, SB and MA-M. Funding acquisition, MA-M. Methodology, MC, SB, and MA-M. Supervision, AA, AI and MA-M. Validation, AA and AI. Writing—original draft, SB. Writing—review and editing, AM\A and HA-M. All authors contributed to the article and approved the submitted version.

## Funding

This study was funded by the Qatar National Research Fund (QNRF) grant number NPRP1556-3-313.

## Conflict of Interest

The authors declare that the research was conducted in the absence of any commercial or financial relationships that could be construed as a potential conflict of interest.
