# Supplementary material for: Genetic Diversity and Prevalence of Giardia duodenalis in Qatar
Source: Front Cell Infect Microbiol. 2021 May 4;11:652946. doi: 10.3389/fcimb.2021.652946 (PMC8129557; doi:10.3389/fcimb.2021.652946)
Supplement: Supplementary file 1 [file Table_1.doc]

***Supplementary table 1: Participant’s demographic data and genetic characterization of their Giardia duodenalis assemblages***

| **Sample ID** | ***Demographic data*** | | | ***Genotyping*** | | | |
| --- | --- | --- | --- | --- | --- | --- | --- |
|  | **Age**  **(year)** | **Gender** | **Nationality** | ***gdh^*^*** | ***bg*** | ***tpi*** | ***MLG***  ***(gdh+bg+tpi)*** |
| **S1** | 30 | F | Indonesian | B | BIII | B | B |
| **S2** | 34 | M | Nepal | B | BIII | BIII | B |
| **S3** | 36 | M | Philippine | B | B | B | B |
| **S4** | 26 | M | Bangladesh | B | BIII | BIII | B |
| **S5** | 29 | M | Nepal | B | B | BIII | B |
| **S6** | 33 | M | Nepal | B | B | A2 | Mixed (A+B) |
| **S7** | 44 | F | Sri Lanka | A2 | B | A2 | Mixed (A+B) |
| **S8** | 30 | M | India | B | B | A2 | Mixed (A+B) |
| **S9** | 27 | M | Bangladesh | B | BIII | BIII | B |
| **S10** | 30 | M | India | B | B | BIII | B |
| **S11** | 21 | M | Nepal | B | BIII | BIII | B |
| **S12** | 21 | M | India | B | BIV | B | B |
| **S13** | 21 | M | India | B | B | A2 | Mixed (A+B) |
| **S14** | 23 | M | Bangladesh | B | BIV | B | B |
| **S15** | 37 | M | Nepal | A2 | A2 | New A2 | New AII |
| **S16** | 21 | M | India | A2 | B | A2 | Mixed (A+B) |
| **S17** | 40 | M | Sri-Lanka | A2 | A2 | A2 | AII-1 |
| **S18** | 43 | M | Nepal | NA | BIV | A2 | Mixed (A+B) |
| **S19** | 19 | M | Nepal | B | B | BIII | B |
| **S20** | 40 | M | India | B | B | BIII | B |
| **S21** | 26 | M | Nepal | B | B | B | B |
| **S22** | 27 | M | Nepal | A2 | A2 | NA | - |
| **S23** | 36 | M | India | A2 | BIII | A2 | Mixed (A+B) |
| **S24** | 25 | M | Nepal | NA | BIV | BIII | - |
| **S25** | 28 | F | Philippine | A2 | A2 | A2 | AII-1 |
| **S26** | - | - | - | A2 | A2 | A2 | AII-1 |
| **S27** | 26 | M | Nepal | A2 | B | A2 | Mixed (A+B) |
| **S28** | 24 | M | Nepal | A2 | A2 | A2 | AII-1 |
| **S29** | 30 | M | Bangladesh | B | B | BIII | B |
| **S30** | 23 | M | India | B | B | BIII | B |
| **S31** | 28 | M | India | B | B | B | B |
| **S32** | 24 | M | Bangladesh | B | B | BIII | B |
| **S33** | 24 | M | Ethiopia | A2 | A2 | A2 | AII-1 |
| **S34** | 30 | M | Ghana | B | A2 | B | Mixed (A+B) |
| **S35** | 26 | M | Nepal | B | B | BIII | B |
| **S36** | 24 | M | India | A2 | A2 | A2 | AII-1 |
| **S37** | 20 | M | Bangladesh | B | BIV | BIII | B |
| **S38** | 25 | M | Bangladesh | B | B | BIII | B |
| **S39** | 22 | M | Bangladesh | A2 | B | A2 | Mixed (A+B) |
| **S40** | 30 | M | India | A2 | BIII | A2 | Mixed (A+B) |
| **S41** | 21 | M | Sri-Lanka | B | BIII | B | B |
| **S42** | 26 | M | India | B | BIII | BIII | B |
| **S43** | 25 | M | India | B | BIV | BIII | B |
| **S44** | 26 | M | Bangladesh | B | B | NA | - |
| **S45** | 26 | M | Bangladesh | B | A2 | B | Mixed (A+B) |
| **S46** | 28 | M | Bangladesh | B | BIII | BIII | B |
| **S47** | 40 | F | India | B | B | BIII | B |
| **S48** | 34 | M | India | B | B | A2 | Mixed (A+B) |
| **S49** | 23 | M | Bangladesh | B | B | A2 | Mixed (A+B) |
| **S50** | 36 | M | Nepal | B | BIII | NA | - |
| **S51** | 28 | M | Nepal | B | B | BIII | B |
| **S52** | 26 | M | Nepal | A2 | A2 | New A2 | New AII |
| **S53** | 25 | M | Nepal | B | B | B | B |
| **S54** | 23 | M | Nepal | B | BIII | B | B |

M: Male, F: Female, NA: not amplified, -: undetermined, ^*^: all assemblage B isolates showed a novel substitution (BIII_JQ700429.1, 212C>T)
